# Efficient DNA knock-in using AAV-mediated delivery with 2-cell embryo CRISPR-Cas9 electroporation

**DOI:** 10.3389/fgeed.2023.1256451

**Published:** 2023-08-25

**Authors:** Daniel J. Davis, James F. McNew, Hailey Maresca-Fichter, Kaiwen Chen, Bhanu P. Telugu, Elizabeth C. Bryda

**Affiliations:** ^1^ Department of Veterinary Pathobiology, University of Missouri, Columbia, MO, United States; ^2^ Comparative Medicine Program, University of Missouri, Columbia, MO, United States; ^3^ School of Veterinary Medicine, Michigan State University, East Lansing, MI, United States; ^4^ School of Veterinary Medicine, Kansas State University, Manhattan, KS, United States; ^5^ Department of Animal Sciences, University of Missouri, Columbia, MO, United States; ^6^ Rat Resource and Research Center, Columbia, MO, United States

**Keywords:** adeno-associated virus (AAV), CRISPR, electroporation, genome editing, 2-cell embryo, knock-in

## Abstract

Recent advances in CRISPR-Cas genome editing technology have been instrumental in improving the efficiency to produce genetically modified animal models. In this study we have combined four very promising approaches to come up with a highly effective pipeline to produce knock-in mouse and rat models. The four combined methods include: AAV-mediated DNA delivery, single-stranded DNA donor templates, 2-cell embryo modification, and CRISPR-Cas ribonucleoprotein (RNP) electroporation. Using this new combined approach, we were able to produce successfully targeted knock-in rat models containing either Cre or Flp recombinase sequences with knock-in efficiencies over 90%. Furthermore, we were able to produce a knock-in mouse model containing a Cre recombinase targeted insertion with over 50% knock-in efficiency directly comparing efficiencies to other commonly used approaches. Our modified AAV-mediated DNA delivery with 2-cell embryo CRISPR-Cas9 RNP electroporation technique has proven to be highly effective for generating both knock-in mouse and knock-in rat models.

## Introduction

Genetically modified animal models are invaluable resources for investigating basic gene function as well as modeling human development, physiology, and disease. In particular, complex animal models with targeted DNA insertions or substitutions (knock-ins) are essential for a variety of applications. In many cases these knock-ins are required to be large in size, which has been shown to reduce gene editing efficiency and limit capabilities of successfully creating the desired model (Lau et al., 2020). CRISPR-Cas technology has widely replaced traditional approaches such as embryonic stem cell targeting for genetically engineering animal models. Numerous technical refinements have been developed to increase CRISPR-Cas mediated genome editing pertaining to creating targeted knock-ins via the homology-directed repair (HDR) pathway. The use of linearized double-stranded DNA (dsDNA) repair templates, single-stranded DNA (ssDNA) repair templates, and chemically modified DNA repair templates have all been used in attempts to increase HDR efficiencies to generate animal models (Gutschner et al., 2016; Zhang et al., 2017; Miura et al., 2018). In addition to altering the DNA repair template properties, optimizing the delivery system has also been attempted to try and increase knock-in efficiencies. Strategies to accomplish this have included pronuclear microinjection along with HDR stimulating compounds, applying electrical pulses in conjunction with microinjection, and timed microinjection into 2-cell embryos (Gu et al., 2018; Gu et al., 2020; Kurihara et al., 2020). While many of these approaches have proven to be successful, the strategies for inserting larger (>1.0 kb) DNA sequences all include technically challenging and low throughput microinjection techniques.

It has been shown that embryo electroporation is equal to or even more efficient than pronuclear microinjection for delivering CRISPR-Cas9 ribonucleoproteins (RNPs) (Alghadban et al., 2020). In addition to being efficient, embryo electroporation is a high-throughput method and does not require technically challenging procedures. However, while it is common to use embryo electroporation to introduce small (<200 bp) ssDNA repair templates, there are limited studies successfully electroporating in larger (>1.0 kb) repair templates (Miyasaka et al., 2018; Teixeira et al., 2018). This size restriction limits our ability to use embryo electroporation for producing knock-in animal models with larger desired insertions.

Adeno-associated viruses (AAVs) have been used for years as genetic modification vehicles due to efficient *in vivo* infectivity, non-pathogenicity, rare genomic integration, and their ability to infect and persist in non-dividing cells (Gaj et al., 2016; Epstein and Schaffer, 2017). However, only recently have AAVs been used in combination with CRISPR-Cas9 technology to introduce DNA repair templates and CRISPR reagents (Mizuno et al., 2018; Chen et al., 2019). This approach offers the unique ability to introduce larger DNA repair templates without the need for microinjection techniques. To build on this work, we modified the AAV approach to be used with embryo electroporation at the 2-cell stage in order to introduce CRISPR-Cas9 RNPs at a more optimal time for engineering targeted DNA insertions. The HDR pathway is predominantly more active in the late S and G2 phases of the cell cycle (Takata et al., 1998; Smirnikhina et al., 2022). It has been shown through *in vitro* studies that timely delivery of CRISPR-Cas9 RNPs and DNA repair templates into G2-synchronized cells or restriction of the presence of Cas9 protein to late S and G2 phases by fusing it with Geminin has been found to significantly increase knock-in efficiency (Gutschner et al., 2016). Moreover, there is a major zygotic genome activation (ZGA) event which takes place during the extended G2 phase of the 2-cell stage embryo. This ZGA event is associated with an open chromatin state and thus likely increases the accessibility of the genomic DNA to CRISPR-Cas9 RNPs and repair templates.

## Results

### Efficiency of modifying 2-cell stage embryos and rationale for timing

**FIGURE 1:**
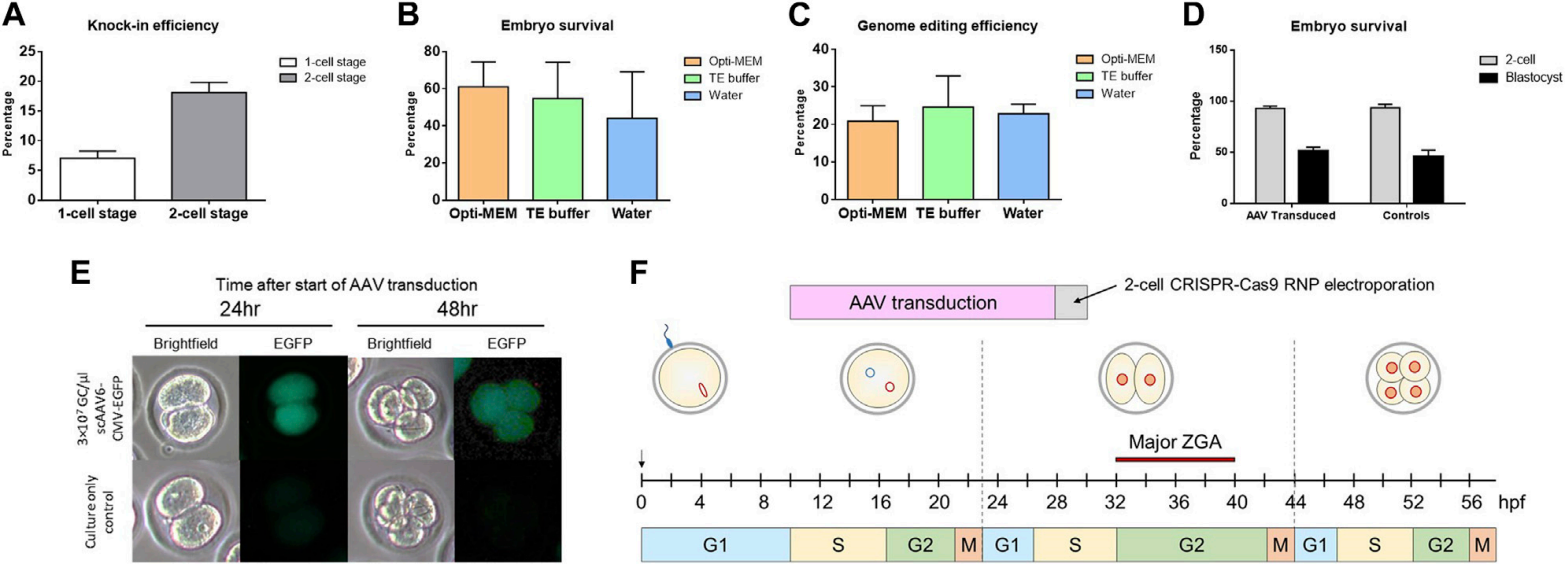
Efficiency of modifying 2-cell stage embryos and rationale for timing. **(A)** Percentage of knock-in positive blastocysts after electroporation at a 1-cell stage or 2-cell stage. Data shown as mean ± SEM of 3 replicate experiments (N = 58 1-cell and N = 34 2-cell embryos analyzed). **(B)** Percentage of embryos to survive to blastocyst stage after electroporation in either Opti-MEM™, TE buffer, or water. Data shown as mean ± SEM of 3 replicate experiments (N = 90 embryos treated per group). **(C)** Percentage of knock-in positive blastocysts after electroporation in either Opti-MEM™, TE buffer, or water. Data shown as mean ± SEM of 3 replicate experiments (number of embryos analyzed in each group = 48 (Opti-MEM™), 42 (TE buffer), and 34 (water)). **(D)** Percentage of embryos to survive until 2-cell stage and blastocyst stage after 18–20 h of AAV transduction. Data shown as mean ± SEM of 3 replicate experiments (N = 65 AAV transduced and N = 52 control embryos analyzed). **(E)** EGFP expression visualized by fluorescence microscopy of 2-cell and 4-cell stage embryos after AAV transduction. **(F)** Schematic showing the timeline of AAV transduction and 2-cell embryo electroporation. DNA was delivered prior to CRISPR reagents in order for the repair template to be present before introducing a double-stranded DNA break. The timing was optimizing for HDR to be able to occur during the longer G2 phase noted in 2-cell embryos with the chromatin being more accessible.

To test whether introduction of CRISPR reagents at a 2-cell stage had an effect on knock-in efficiency, we electroporated Sprague Dawley (NTac:SD) embryos at either a 1-cell stage or a 2-cell stage with CRISPR sgRNA/Cas9 RNPs along with a 200 bp ssDNA repair template. The DNA repair template had 35 bp homology arms and was designed to insert a unique 130 bp sequence into the rat genome. A 157% increase in knock-in efficiency was detected in embryos that had reagents delivered at the 2-cell stage compared to the 1-cell stage (Figure 1A). We also tested to see if the buffer used during electroporation had an impact on embryo survival or genome editing efficiency (Figures 1B,C). While we did not note any significant difference between using Opti-MEM™, TE buffer or water, Opti-MEM™ was used in all subsequent electroporations. For AAV experiments, we chose a final viral concentration of 3×10^7^ viral genome copies (GC)/µl with the rationale that it is within the effective dose range shown in previously published work (1 × 10^7^ GC/μL to 4 × 10^8^GC/μL) and lower than the doses shown to have toxicity effects (1 × 10^9^GC/μL) (Mizuno et al., 2018; Chen et al., 2019). Furthermore, AAV serotype 6 was chosen based on its known efficiency for gene delivery into mouse embryos (Mizuno et al., 2018; Yoon et al., 2018; Chen et al., 2019). To assess AAV-mediated DNA delivery timing, we wanted to investigate if embryos could be infected with virus at a 1-cell stage and cultured overnight to 2-cell stage without a deleterious effect on embryo development. scAAV6-CMV-EGFP was added to 1-cell stage Sprague Dawley (NTac:SD) embryos at a concentration of 3 × 10^7^GC/μL. We noted no significant differences in embryo development or survival compared to non-infected controls (development to 2-cell embryos = 93.0% for AAV transduced and 93.5% for controls; survival to blastocyst stage = 51.5% for AAV transduced and 46.0% for controls) (Figure 1D) and we detected robust EGFP expression in the embryos infected with scAAV6-CMV-EGFP (Figure 1E). These results guided development of a modified pipeline to introduce a DNA repair template via AAV-mediated delivery at the 1-cell stage and then electroporate CRISPR sgRNA/Cas9 RNPs at the 2-cell stage in order to allow for optimal timing of the DNA knock-in through homology-directed repair during the longer G2 phase (Figure 1F).

### Generation of knock-in rat models using optimized pipeline

**FIGURE 2:**
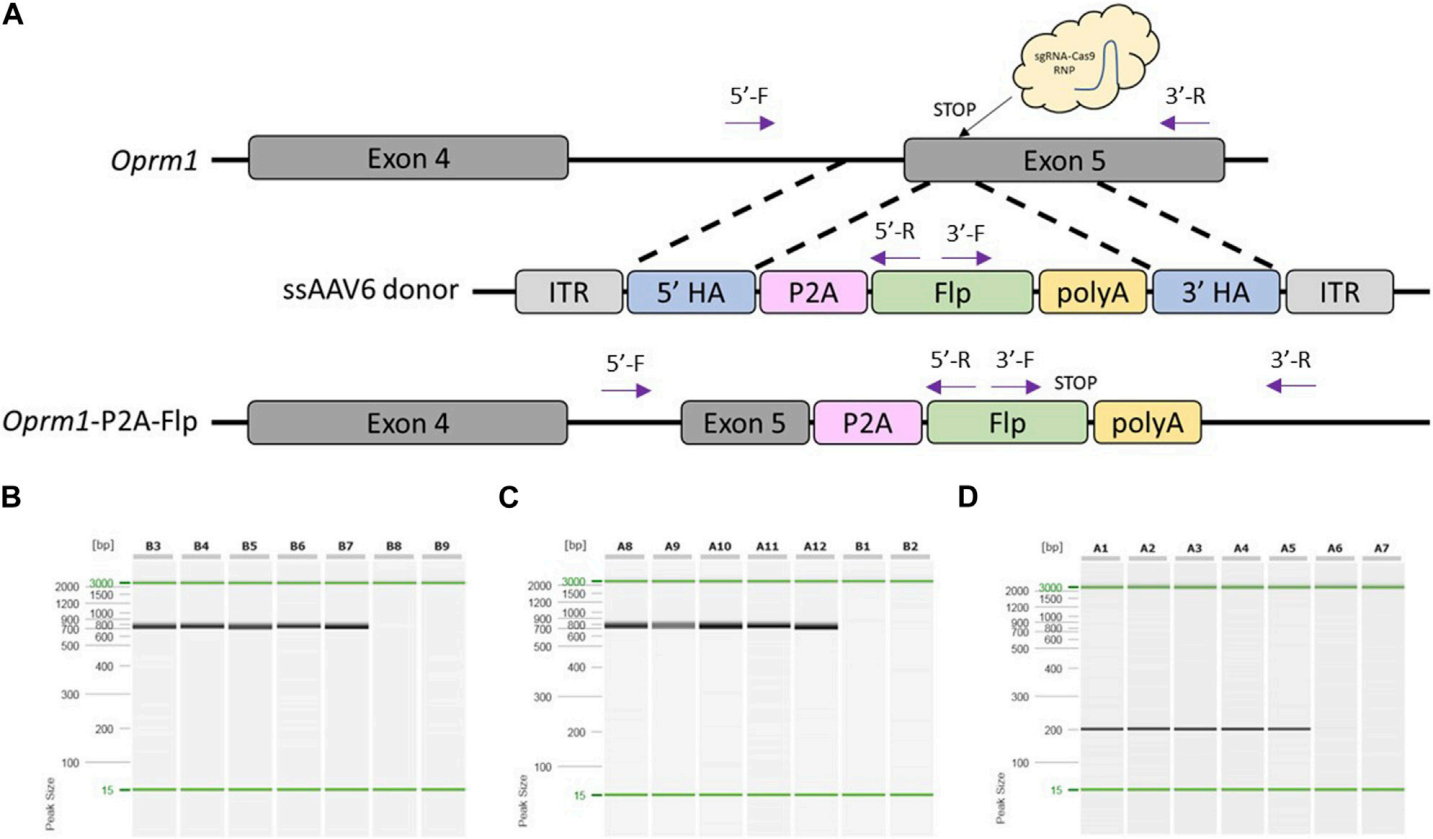
Generation of *Oprm1*-P2A-Flp knock-in rat model using optimized pipeline. **(A)** Schematic of rat *Oprm1* locus and gene targeting strategy. PCR primer locations noted by arrows illustrating PCR assays extend across each homology arm. **(B)** PCR across 5′homology arm. Lanes B3-B7 contain DNA samples from founder animals, lane B8 contains DNA sample from a WT control rat, and lane B9 is a no DNA template control. **(C)** PCR across 3′homology arm. Lanes A8-A12 contain DNA samples from founder animals, lane B1 contains DNA sample from a WT control rat, and lane B2 is a no DNA template control. **(D)** Flp-specific internal PCR. Lanes A1-A5 contain DNA samples from founder animals, lane A6 contains DNA sample from a WT control rat, and lane A7 is a no DNA template control. PCR reactions were analyzed using a QIAxcel Advanced capillary electrophoresis system with a 15bp-3kb alignment marker (denoted by the green lines) and QX DNA size marker. Peak sizes correspond to PCR amplicon lengths.

To test if our modified pipeline was efficient to produce a knock-in rat model, we designed a project to target a P2A-Flp cassette that replaced the endogenous STOP codon of rat *Oprm1* (Figure 2A). Sprague Dawley (NTac:SD) embryos were collected at a 1-cell stage, infected with 3 × 10^7^GC/μL ssAAV6-5′ homology arm-P2A-Flp-3′ homology arm for 18–20 h, and electroporated with a CRISPR sgRNA/Cas9 RNP targeted to the rat *Oprm1* STOP codon. Of the offspring born, 100% (5/5) were found to have the desired P2A-Flp targeted knock-in based on PCR amplification across each homology arm and PCR amplification with primers internal to the Flp cassette (Figures 2B–D). The P2A-Flp insertion of these founder animals was confirmed by Sanger sequence analysis across each homology arm (Supplementary Material). Two founder animals were bred to wild type mates and both lines successfully transmitted the desired knock-in allele to their offspring. For the first line, 52.6% (10/19) offspring were heterozygous for the desired knock-in allele and 47.4% (9/19) contained only the wild type allele. These were the only two alleles detected in offspring for the first line. For the second line, 34.8% (8/23) offspring were heterozygous for the desired knock-in allele, 43.5% (10/23) were heterozygous for a partial knock-in allele, and 21.7% (5/23) contained only the wild type allele. These results suggest a degree of mosaicism in some founder animals which is common to most approaches using CRISPR-mediated genome editing in embryos.

**FIGURE 3:**
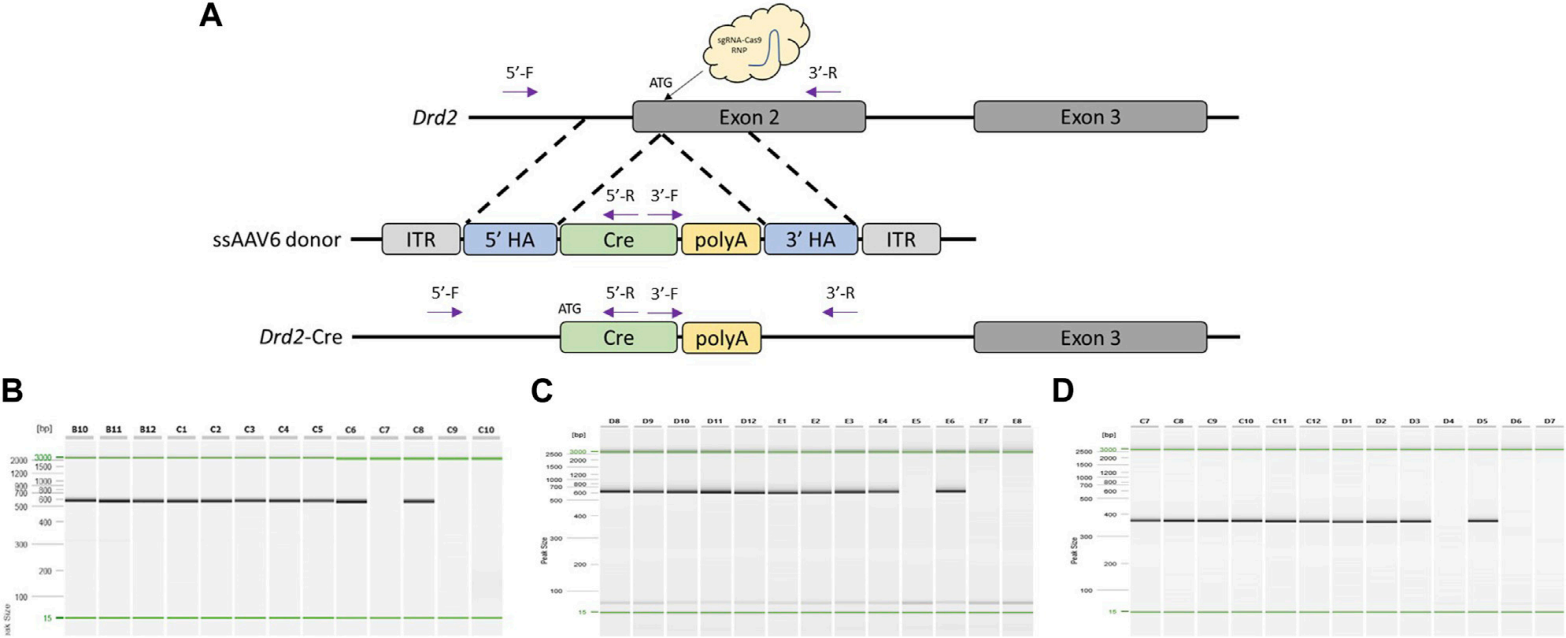
Generation of *Drd2*-Cre knock-in rat model using optimized pipeline. **(A)** Schematic of rat *Drd2* locus and gene targeting strategy. PCR primer locations noted by arrows illustrating PCR assays extend across each homology arm. **(B)** PCR across 5′homology arm. Lanes B10-C8 contain DNA samples from founder animals, lane C9 contains DNA sample from a WT control rat, and lane C10 is a no DNA template control. **(C)** PCR across 3′homology arm. Lanes D8-E6 contain DNA samples from founder animals, lane E7 contains DNA sample from a WT control rat, and lane E8 is a no DNA template control. **(D)** Cre-specific internal PCR. Lanes C7-D5 contain DNA samples from founder animals, lane D6 contains DNA sample from a WT control rat, and lane D7 is a no DNA template control. PCR reactions were analyzed using a QIAxcel Advanced capillary electrophoresis system with a 15bp-3kb alignment marker (denoted by the green lines) and QX DNA size marker. Peak sizes correspond to PCR amplicon lengths.

To test another locus and knock-in sequence with our modified delivery pipeline, we designed a project to target a Cre cassette to the ATG start site of rat *Drd2* (Figure 3A). Sprague Dawley (NTac:SD) embryos were collected at a 1-cell stage, infected with 3 × 10^7^ GC/μL ssAAV6-5′ homology arm-Cre-3′ homology arm for 18–20 h, and electroporated with a CRISPR sgRNA/Cas9 RNP targeted to the rat *Drd2* ATG start site. Of the offspring born, 91% (10/11) were found to have the desired Cre targeted knock-in based on PCR amplification across each homology arm and amplification using primers internal to the Cre cassette (Figures 3B–D) as well as confirmation by Sanger sequence analysis across each homology arm (Supplementary Material). Similar to the *Oprm1*-P2A-Flp model, two founders were bred to wild type mates and both lines successfully transmitted the desired knock-in allele to their offspring. However, in this case both founder lines produced 100% (8/8 and 21/21, respectively) offspring heterozygous for the desired knock-in allele suggesting that both founders bred were homozygous for the knock-in allele.

### Generation of knock-in mouse model and direct comparison with other approaches

**FIGURE 4:**
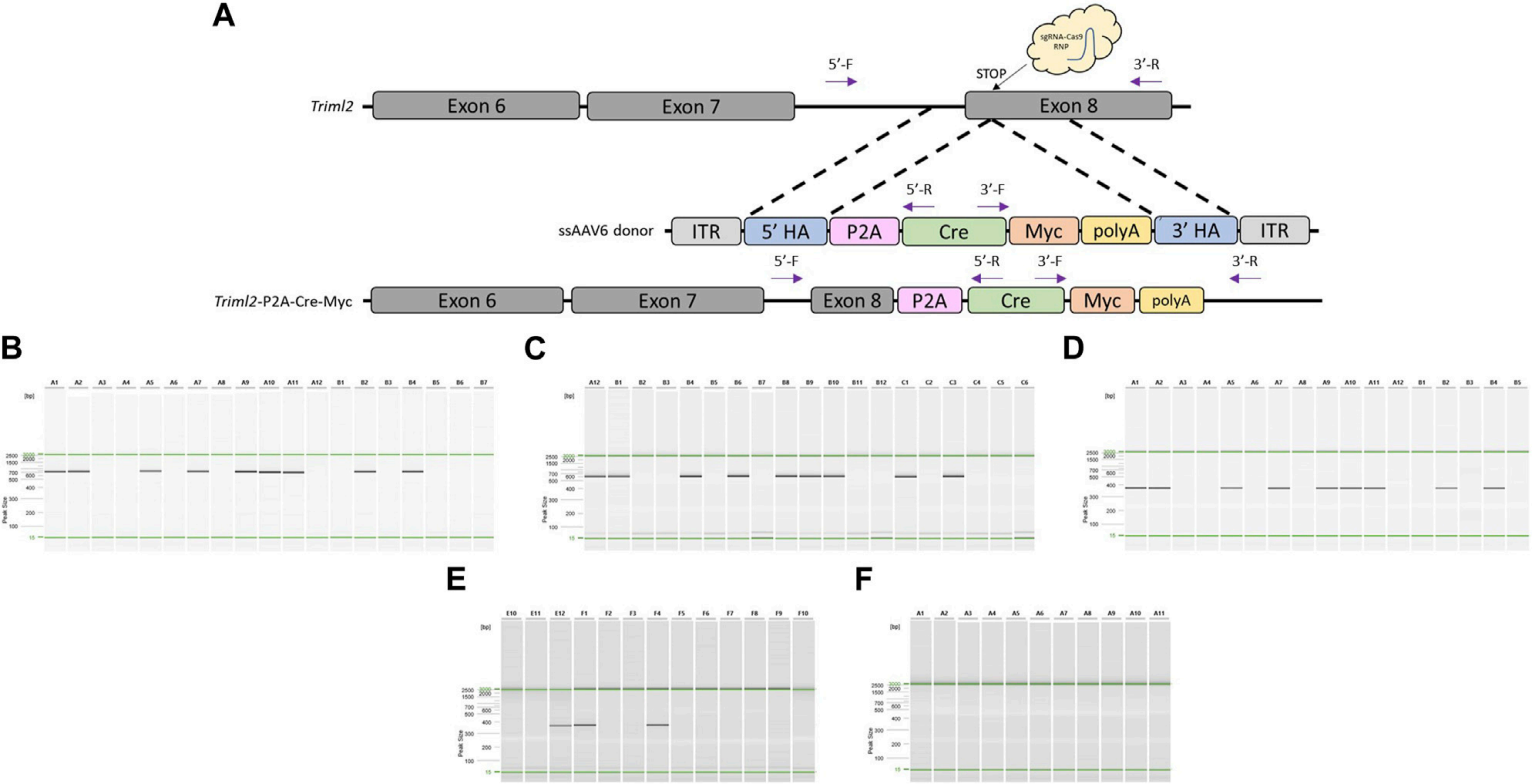
Generation of *Triml2*-P2A-Cre-Myc knock-in mouse model using optimized pipeline. **(A)** Schematic of mouse *Triml2* locus and gene targeting strategy. PCR primer locations noted by arrows illustrating PCR assays extend across each homology arm. **(B)** PCR across 5′homology arm. Lanes A1-B5 contain DNA samples from founder animals, lane B6 contains DNA sample from a WT control rat, and lane B7 is a no DNA template control. **(C)** PCR across 3′homology arm. Lanes A12-C4 contain DNA samples from founder animals, lane C5 contains DNA sample from a WT control rat, and lane C6 is a no DNA template control. **(D)** Cre-specific internal PCR. Lanes A1-B5 contain DNA samples from founder animals, *Note: WT and no DNA template controls were not included in this assay.* **(E)** Cre-specific internal PCR for AAV + 1-cell electroporation approach. Lanes E10-F8 contain DNA samples from founder animals, lane F9 contains DNA sample from a WT control rat, and lane F10 is a no DNA template control. **(F)** Cre-specific internal PCR for dsDNA Alt-R™ HDR Donor Block PNI approach. Lanes A1-A9 contain DNA samples from founder animals, lane A10 contains DNA sample from a WT control rat, and lane A11 is a no DNA template control. PCR reactions were analyzed using a QIAxcel Advanced capillary electrophoresis system with a 15bp-3kb alignment marker (denoted by the green lines) and QX DNA size marker. Peak sizes correspond to PCR amplicon lengths.

Once we had demonstrated that this modified pipeline was efficient to generate knock-in rat models, we tested the technique for the generation of a mouse model. Moreover, we decided to make a direct comparison of this method on a project that had already proven to be difficult using an alternative knock-in approach. This project was designed to replace the endogenous STOP codon of mouse *Triml2* with a P2A-Cre cassette (Figure 4A). C57BL/6J embryos were collected at a 1-cell stage, infected with 3 × 10^7^GC/μL ssAAV6-5′ homology arm-P2A-Cre-3′ homology arm for 18–20 h, and electroporated with a CRISPR sgRNA/Cas9 RNP targeted to the mouse *Triml2* STOP codon. Of the offspring born, 56% (9/16) were found to have the desired P2A-Cre targeted knock-in based on PCR amplification across each homology arm and PCR amplification using primers located within the Cre cassette (Figures 4B–D) as well as confirmation by Sanger sequence analysis across each homology arm (Supplementary Material). To compare our 2-cell electroporation method to the traditional 1-cell electroporation approach, C57BL/6J embryos were collected at a 1-cell stage, infected with 3 × 10^7^ GC/μL ssAAV6-5′ homology arm-P2A-Cre-3′ homology arm for 6 h, and then electroporated with the same CRISPR sgRNA/Cas9 RNP. Of the offspring born, 27% (3/11) were found to have the desired P2A-Cre targeted knock-in (Figure 4E). Lastly, using the same CRISPR sgRNA/Cas9 RNP and a dsDNA 5′homology arm-P2A-Cre-3′ homology arm (Alt-R™ HDR Donor Block) along with pronuclear injections at the 1-cell stage, we were not able to detect any positive offspring (0/8) (Figure 4F). Note, the homology arms in the dsDNA Alt-R™ HDR Donor Block were the same as used in the ssAAV6 constructs (5′homology arm = 482bp and 3′homology arm = 444bp).

## Discussion

Because of the versatility of the popular CRISPR-Cas genome editing system, there are frequent refinements and improvements in techniques used for generating custom genetically modified animal models allowing for more efficient production of complex models. Some of the most promising refinements include the use of ssDNA templates, zygote electroporation, 2-cell embryo manipulation, and AAV-mediated DNA delivery. Here we have combined these promising techniques and demonstrated that using ssAAV-mediated DNA delivery along with 2-cell embryo electroporation of CRISPR-Cas9 reagents is an effective way for producing targeted knock-in animal models.

Through our direct comparison of using dsDNA Alt-R™ HDR Donor Block *versus* ssAAV6 construct, we were able to repeat what others have shown in that ssDNAs (whether delivered as naked ssDNA or ssAAV capsulated) yield higher targeted insertion efficiencies (Miura et al., 2015; Quadros et al., 2017; Codner et al., 2018; Miura et al., 2018). It is speculated that dsDNA donor templates use classic homologous recombination as a homology-directed repair (HDR) mechanism, while ssDNA donor templates may rely on the proteins involved with single strand annealing and micro-homology mediated end joining (Zhang et al., 2022). This leads to the question of whether the ssAAV viral capsulation further aides in delivering the ssDNA donor template to the nucleus more effectively than naked ssDNA or if the inverted terminal repeat (ITR) sequences on the ssAAV construct are responsible for increasing efficiency. To that end, it has been shown previously that ITR sequences are important for intermolecular homologous recombination and circularization of AAV genomes (Yan et al., 2005). This could be tested by introducing non-capsulated naked ssDNA that contains ITR sequences in comparison to naked ssDNA alone.

In addition to using ssDNA, our 2-cell embryo electroporation approach demonstrated increased knock-in efficiencies compared to 1-cell embryo electroporation. This is presumably due to the open chromatin state during genome activation and the fact that HDR is predominantly active in the late S-G2 phases (Gu et al., 2018; Plaza Reyes and Lanner, 2018). We speculate that the increased knock-in efficiency observed in our 2-cell embryo electroporation approach is related to the timing of electroporation rather than the length of AAV infection time since there have previously been systematic experiments showing no increase in embryo AAV transduction with longer incubation times (Chen et al., 2019). While 2-cell embryo electroporation increases knock-in efficiency, it is also important to note that cellular fusion is observed in some 2-cell embryos during the electroporation process. However, this phenomenon is not surprising due to the fact that electrofusion procedures are commonly used for fusing mammalian cells (Fulka et al., 1995).

Aside from differences in incubation times, some other groups have thinned the zona pellucida before infecting embryos with AAV (Mizuno et al., 2018; Chen et al., 2019). The zona pellucida is a hardened glycoprotein matrix surrounding the fertilized egg and often complicates nucleic acid delivery by forming a physical barrier against viruses and transfection reagents. However, it has been shown recently that some serotypes of AAV are able to diffuse across the zona pellucida without the need for thinning or disruption (Romeo et al., 2020). To this point, the experiments described here were performed without thinning the zona pellucida. It is plausible that we did not see a detrimental effect of higher AAV concentrations due to the zona pellucida not being thinned in comparison to previous studies that noted decreased embryo development with higher AAV concentrations (Chen et al., 2019).

AAV-mediated DNA delivery is an elegant complement to our 2-cell embryo electroporation technique. With electroporation alone, we and others have noted that there appears to be a size limitation to the DNA template being able to effectively enter the nucleus which is required for integration into the genome. AAV-mediated DNA delivery permits us to still take advantage of the benefits of 2-cell embryo electroporation while increasing the ssDNA donor template size to 4.3 kb. This increase in the donor DNA size limitation allows for the generation of several types of complex animal models including conditional knockouts, reporters, humanized gene replacements, and more. Future breakthroughs to increase AAV package size restrictions or the use of other viral and non-viral mediated DNA delivery approaches can easily be adapted to work in conjunction with 2-cell embryo electroporation. In conclusion, combining the use of ssAAV6-mediated DNA delivery and 2-cell embryo electroporation of CRISPR sgRNA/Cas9 RNPs allows for efficient generation of knock-in rat and mouse models.

## Methods

### Animals

All experimental procedures were approved by the University of Missouri’s Institutional Animal Care and Use Committee and were performed according to the guidelines set forth in the Guide for the Use and Care of Laboratory Animals. For production of rat embryos, NTac:SD females (4 weeks of age) and NTac:SD males (10 weeks of age) were purchased from Taconic Biosciences. For production of mouse embryos, C57BL/6J females (4 weeks of age) and C57BL/6J males (10 weeks of age) were purchased from the Jackson Laboratory. For rat embryo transfers, NTac:SD females (8 weeks of age) were purchased from Taconic Biosciences. For mouse embryo transfers, CD-1 females (8 weeks of age) were purchased from Charles River Laboratories. All animals were housed in ventilated cages (Thoren) and kept on a 12:12 light cycle. Food and water were available *ad libitum*.

#### Superovulation and embryo collection

To collect 1-cell stage embryos (zygotes) from immature (4 weeks old) female NTac:SD rats, females were superovulated by intraperitoneal administration of 25 IU pregnant mare serum gonadotropin (PMSG) followed by intraperitoneal administration of 40 IU human chorionic gonadotropin (hCG) 50 h later. Females were then immediately co-housed with NTac:SD stud males (10+ weeks of age) to allow mating. Zygotes were collected at 22–24 h after hCG administration from copulation plug-positive females. After CO_2_ euthanasia, oviducts were excised, and cumulus enclosed zygotes were released from the oviduct by tearing the swollen ampulla using fine forceps and an insulin needle. Zygotes were then denuded (removal of cumulus cells) by briefly exposing to 1 mg/mL hyaluronidase in mFHM and washed in mFHM +4 mg/mL fatty acid free BSA.

Similarly, to collect zygotes from immature (4 weeks old) female C57BL/6J mice, females were superovulated by intraperitoneal administration of 5 IU PMSG followed by intraperitoneal administration of 5 IU hCG 48 h later. Females were then mated to C57BL/6J stud males (10+ weeks of age). Zygotes were collected at 22–24 h after hCG administration from copulation plug-positive females. After CO_2_ euthanasia, oviducts were excised, and cumulus enclosed zygotes were released from the oviduct by tearing the swollen ampulla using fine forceps and an insulin needle. Zygotes were then denuded by briefly exposing to 1 mg/mL hyaluronidase in mFHM and washed in mFHM +4 mg/mL fatty acid free BSA.

#### AAV-mediated DNA delivery and embryo culture

For EGFP experiments, SD:NTac zygotes with visible pronuclei were placed in 30 μL KSOM-R medium (Men et al., 2023) containing 3 × 10^7^GC/μL scAAV6-CMV-EGFP (VectorBuilder) under mineral oil in 35 mm Petri dish and cultured at 37°C with 5% CO_2_ and maximal humidity for 18–20 h. The following day 2-cell stage embryos were washed 3 times in KSOM-R and moved to 500 μL KSOM-R for further culture. Embryos were imaged daily using a Nikon Eclipse TS100 microscope and Lumencor^®^ Sola light engine (Ex/Em = 488/507 nm). Embryo development and survival rates were assessed daily until the blastocyst stage.

For animal model generation, zygotes with visible pronuclei were placed in 30 μL medium (KSOM for mouse embryos and KSOM-R for rat embryos (Men et al., 2023)) containing 3 × 10^7^ GC/μL ssAAV6 (VectorBuilder) under mineral oil in 35 mm Petri dish and cultured at 37°C with 5% CO_2_ and maximal humidity for 18–20 h. The following day 2-cell stage embryos were electroporated with CRISPR sgRNA/Cas9 RNP complexes. After electroporation, the 2-cell stage embryos were transferred to 1.5-day post coitum (dpc) surrogate females.

#### CRISPR-Cas9 RNP electroporation

The CRISPR RGEN Tools website (http://www.rgenome.net) maintained by the Center for Genome Engineering Institute (Korea) was used to calculate off target scores and design sgRNAs. The CCTop website (https://cctop.cos.uni-heidelberg.de:8043/) maintained by the Centre for Organismal Studies (Heidelberg) was used to calculate CRISPRater efficiency prediction scores for each sgRNA (Stemmer et al., 2015; Labuhn et al., 2018). sgRNA was ordered as a chemically modified synthetic sgRNA through Synthego. The chemical modifications were 2′-O-methyl analogs and 3′phosphorothioate internucleotide linkages at the first three 5′and 3′terminal RNA residues.

RNP complexes were formed by mixing 100 ng/μL sgRNA +100 ng/μL Cas9 protein with Opti-MEM™ as buffer in the reagent mix and incubating at room temperature for 10 min. Note, that for the electroporation media testing experiment in Figure 1, TE buffer or water was substituted for Opti-MEM™ as buffer in the reagent mix. Using a NepaGene21 super electroporator and a 1 mm gap glass slide electrode, 5–6 µL of sgRNA/Cas9 RNP mix were loaded into the electrode. Embryos (20–40) were then loaded s quickly, keeping them toward the middle of electrode without touching sides. The impedance was checked. The volume was adjusted as needed so that the impedance was between 0.100 and 0.300 Ω. Embryos were electroporated under the following conditions: Poring pulse: 40V, 3.5 ms length, 50 ms interval, 10% decay rate, positive polarity (x4 pulses). Transfer pulse: 5V, 50 ms length, 50 ms interval, 40% decay rate, alternating polarity (x5 pulses). Embryos were removed from the electrode immediately following electroporation and cultured *in vitro*.

#### Model genetic characterization

The *Oprm1*-P2A-Flp model was generated using the above methods with a ssAAV6 vector containing a P2A-Flp cassette flanked by homology arms (5′= 517 bp and 3′= 536) corresponding to the expected dsDNA break using an *Oprm1* targeted sgRNA: 5′GAT​GGT​GTG​AGA​CCC​AGT​TA 3′. At 2 weeks of age, tail biopsy samples were taken, and DNA extracted using a Qiagen DNeasy Blood and Tissue kit according to manufacturer’s instructions. The *Oprm1*-P2A-Flp model was genotyped using the following primer sets: Assay 1 across 5′homology arm: forward primer 5′TCA​AAG​GGT​GCG​CTC​CAC​AGT​G 3′+ reverse primer 5′CTC​AGG​CTG​TTG​CTG​ATG​ATG​G 3′= 860 bp amplicon; Assay 2 across 3′homology arm: forward primer 5′ACT​ACC​TGA​GCA​GCT​ACA​TCA​ACA​G 3′+ reverse primer 5′CAT​TCT​GCA​GTT​GAC​ACT​GTG​C 3′= 895 bp amplicon; Assay 3 internal FLP cassette: forward primer 5′AGG​AAG​GTG​ATG​AGC​CAG​TTC​G 3′+ reverse primer 5′CTC​AGG​CTG​TTG​CTG​ATG​ATG​G 3′= 215 bp amplicon. All assays were performed using the following PCR parameters: 1) 95 C for 3 min, 2) 95 C for 30 s, 3) 61 C for 30 s, 4) 72 C for 1 min (steps 2–4) × 35, 5) 7 C for 7 min, 6) 4 °C hold.

The *Drd2*-Cre model was generated using the above methods with a ssAAV6 vector containing a Cre cassette flanked by homology arms (5′= 422 bp and 3′= 477) corresponding to the expected dsDNA break using an *Drd2* targeted sgRNA: 5′CAG​GTT​CAG​TGG​ATC​CAT​TG 3′. At 2 weeks of age, tail biopsy samples were taken, and DNA extracted using a Qiagen DNeasy Blood and Tissue kit according to manufacturer’s instructions. The *Drd2*-Cre model was genotyped using the following primer sets: Assay 1 across 5′homology arm: forward primer 5′CCA​GCA​TTT​GGA​GCA​ACT​GGA​G 3′+ reverse primer 5′ACC​TCA​TCA​CTC​GTT​GCA​TCG​AC 3′= 601 bp amplicon; Assay 2 across 3′homology arm: forward primer 5′GCA​TCG​CAT​TGT​CTG​AGT​AGG​TG 3′+ reverse primer 5′CTG​CCA​GAT​GAT​GAC​AGT​GCC 3′= 676 bp amplicon; Assay 3 internal Cre cassette: forward primer 5′AAG​ATA​TCT​CAC​GTA​CTG​ACG​GTG​G 3′+ reverse primer 5′TGA​TCT​CCG​GTA​TTG​AAA​CTC​CAG​C 3′= 381 bp amplicon. All assays were performed using the following PCR parameters. 1) 95 C for 3 min, 2) 95 °C for 30 s, 3) 61 C for 30 s, 4) 72 C for 1 min (steps 2–4) × 35, 5) 7°C for 7 min, 6) 4°C hold.

The *Triml2*-P2A-Cre model was generated using the above methods with a ssAAV6 vector containing a P2A-Cre cassette flanked by homology arms (5′= 482 bp and 3′= 444) corresponding to the expected dsDNA break using an *Triml2* targeted sgRNA: 5′GAG​CTA​TCA​GCA​GCA​TTT​CA 3′. At 3 weeks of age, tail biopsy samples were taken, and DNA extracted using a Qiagen DNeasy Blood and Tissue kit according to manufacturer’s instructions. The *Triml2*-P2A-Cre model was genotyped using the following primer sets: Assay 1 across 5′ homology arm: forward primer 5′CTC​CCT​AGT​CTT​ATC​CGA​AGA​CCT​G 3′+ reverse primer 5′ACC​TCA​TCA​CTC​GTT​GCA​TCG​AC 3′= 651 bp amplicon; Assay 2 across 3′ homology arm: forward primer 5′GTT​GTG​GTT​TGT​CCA​AAC​TCA​TC 3′+ reverse primer 5′CAG​CCC​AAA​TTC​TAG​GTC​TTT​CG 3′= 648 bp amplicon; Assay 3 internal Cre cassette: forward primer 5′AAG​ATA​TCT​CAC​GTA​CTG​ACG​GTG​G 3′+ reverse primer 5′TGA​TCT​CCG​GTA​TTG​AAA​CTC​CAG​C 3′= 381 bp amplicon. All assays were performed using the following PCR parameters. 1) 95 C for 3 min, 2) 95 C for 30 s, 3) 61 C for 30 s, 4) 72 C for 1 min (steps 2–4) × 35, 5) 7 C for 7 min, 6) 4 C hold.

## Data Availability

The raw data supporting the conclusion of this article will be made available by the authors, without undue reservation.
