# Supplementary material for: Efficient DNA knock-in using AAV-mediated delivery with 2-cell embryo CRISPR-Cas9 electroporation
Source: Front Genome Ed. 2023 Aug 25;5:1256451. doi: 10.3389/fgeed.2023.1256451 (PMC10485772; doi:10.3389/fgeed.2023.1256451)
Supplement: Supplementary file 1 [file Table1.DOCX]

***Oprm1*-P2A-Flp raw sequence reads**

Across 5’ homology arm and P2A into Flp:

Animal ID 119DF

CAAAGGGGGGTCGCGTCCACAGTGGGAGGTGAATGGCAGTGTACTCCACACCTCCCACCAGGGGTGAAAACAGGAAGGAATTCTAACAATTGTGTGCAAAATAAAAATGTTTTCATTCTATTTCTTTTTGATATCTTGGTTCAGTCATTATTGAGTTAACTGGAAAGGGAGAACATGGAAGATGGAGCAAGAGAAAGAATTTTACTTAAAATGTATTTTCTAAGGTCTAGTGTGGCATATCTCTGCAATCTTGGTGCCCAAAAAGCTGAGGTAGGAGGATCTCAAATTAAAGCTCGCCCTGGGCTACTGAGCAAAATTCTAGCTAAAGAAAACTAATGTTCATCTTTATAATGAGTACTATATGCCAACACATATTTATGATGGCCCTCATTCTGAGCTCCCAACAAAAGCACAAGACAATGTTCAGTACAGTTCTCATACCCAGAAGACAATCATCAATGTGCTCTCTAATGAGACCCCAGAACTCACTATCTTCACTCTTTCTCTTCTTTCAGCTAGAAAATCTGGAGGCAGAAACTGCTCCATTGCCCGTATACGGAAGCGGAGCTACTAACTTCAGCCTGCTGAAGCAGGCTGGAGACGTGGAGGAGAACCCTGGACCTGCTAGCATGGCTCCTAAGAAGAAGAGGAAGGTGATGAGCCAGTTCGACATCCTGTGCAAGACCCCCCCCAAGGTGCTGGTGCGGCAGTTCGTGGAGAGATTCGAGAGGCCCAGCGGCGAGAAGATCGCCAGCTGTGCCGCCGAGCTGACCTACCTGTGCTGGATGATCACCCACAACGGCACCGCCATCAAGAGGGCCAC-TTCA

Animal ID 120DF

GTCCGCTCCACAGTGGAGGTGAATGGCAGTGTACTCCACACCTCCCACCAGGGGTGAAAACAGGAAGGAATTCTAACAATTGTGTGCAAAATAAAAATGTTTTCATTCTATTTCTTTTTGATATCTTGGTTCAGTCATTATTGAGTTAACTGGAAAGGGAGAACATGGAAGATGGAGCAAGAGAAAGAATTTTACTTAAAATGTATTTTCTAAGGTCTAGTGTGGCATATCTCTGCAATCTTGGTGCCCAAAAAGCTGAGGTAGGAGGATCTCAAATTAAAGCTCGCCCTGGGCTACTGAGCAAAATTCTAGCTAAAGAAAACTAATGTTCATCTTTATAATGAGTACTATATGCCAACACATATTTATGATGGCCCTCATTCTGAGCTCCCAACAAAAGCACAAGACAATGTTCAGTACAGTTCTCATACCCAGAAGACAATCATCAATGTGCTCTCTAATGAGACCCCAGAACTCACTATCTTCACTCTTTCTCTTCTTTCAGCTAGAAAATCTGGAGGCAGAAACTGCTCCATTGCCCGTATACGGAAGCGGAGCTACTAACTTCAGCCTGCTGAAGCAGGCTGGAGACGTGGAGGAGAACCCTGGACCTGCTAGCATGGCTCCTAAGAAGAAGAGGAAGGTGATGAGCCAGTTCGACATCCTGTGCAAGACCCCCCCCAAGGTGCTGGTGCGGCAGTTCGTGGAGAGATTCGAGAGGCCCAGCGGCGAGAAGATCGCCAGCTGTGCCGCCGAGCTGACCTACCTGTGCTGGATGATCACCCACAACGGCACCGCCATCAAGAGG-CCACCT-CA

Animal ID 121DF

TGCGCTCCACAGTGGGAGGTGAATGGCAGTGTACTCCACACCTCCCACCAGGGGTGAAAACAGGAAGGAATTCTAACAATTGTGTGCAAAATAAAAATGTTTTCATTCTATTTCTTTTTGATATCTTGGTTCAGTCATTATTGAGTTAACTGGAAAGGGAGAACATGGAAGATGGAGCAAGAGAAAGAATTTTACTTAAAATGTATTTTCTAAGGTCTAGTGTGGCATATCTCTGCAATCTTGGTGCCCAAAAAGCTGAGGTAGGAGGATCTCAAATTAAAGCTCGCCCTGGGCTACTGAGCAAAATTCTAGCTAAAGAAAACTAATGTTCATCTTTATAATGAGTACTATATGCCAACACATATTTATGATGGCCCTCATTCTGAGCTCCCAACAAAAGCACAAGACAATGTTCAGTACAGTTCTCATACCCAGAAGACAATCATCAATGTGCTCTCTAATGAGACCCCAGAACTCACTATCTTCACTCTTTCTCTTCTTTCAGCTAGAAAATCTGGAGGCAGAAACTGCTCCATTGCCCGTATACGGAAGCGGAGCTACTAACTTCAGCCTGCTGAAGCAGGCTGGAGACGTGGAGGAGAACCCTGGACCTGCTAGCATGGCTCCTAAGAAGAAGAGGAAGGTGATGAGCCAGTTCGACATCCTGTGCAAGACCCCCCCCAAGGTGCTGGTGCGGCAGTTCGTGGAGAGATTCGAGAGGCCCAGCGGCGAGAAGATCGCCAGCTGTGCCGCCGAGCTGACCTACCTGTGCTGGATGATCACCCACAACGGCACCGCCATCAAGAGGGCCACCTT-A

Out of Flp across SV40 poly(A) and 3’ homology arm:

Animal ID 119DF

ACTACCTGAAGCAGCTACATCAACAGGCGGATCTAAATGCATCAGCCATACCACATTTGTAGAGGTTTTACTTGCTTTAAAAAACCTCCCACACCTCCCCCTGAACCTGAAACATAAAATGAATGCAATTGTTGTTGTTAACTTGTTTATTGCAGCTTATAATGGTTACAAATAAAGCAATAGCATCACAAATTTCACAAATAAAGCATTTTTTTCACTGCATTCTAGTTGTGGTTTGTCCAAACTCATCAATGTATCTTACTGGGTCTCACACCATCCAGACCCTCGCTAAGCTTAGAGGCCGCCATCTACGTGGAATCAGGTTGCTGTCAGGGTGTGTGGGAGGCTCTGGTTTCCTGAGAAACCATCTGATCCTGCATTCAAAGTCATTCCTCTCTGGCTACTTCACTCTGCACATGAGAGATGCTCAGACTGTATCAAGTACTCAGAAAGAAGAGACTACCGGACACTCCTGAATCCAGCTCATGTACAGAACCATCTGAAACACCCAGTGGACCACAATGCTCTGTGGTATGTGAATTTCGATCATCATAGAAGGTGACCCCTCTCTATGTAGAATTTTTATTTTTCAAGCAAATACTTATGACCTCATCAAAGAAAATAATGTCACTTGTTAAATTCACTGTAGTGATACATAAAGTAAATGCTACCTCTGACCTCTGACCCAGTCACCTTCTGTAGAGAGTTCCAGTCCTTTTGTGATGGAATACATCATTTCCAACTTAAAACTTTCACCTTGAAGTTATGGTCTAGTTAAGACATCAGGGGCACCTCCGTTTCTTGGTTTTGTATTGTT-GAAAGAAGACGACATCTTCCTCCTTAGCTGTGTGT-GAAAA

Animal ID 120DF

GCAGCTACATCAAACAGGCGGATCTAAATGCATCAGCCATACCACATTTGTAGAGGTTTTACTTGCTTTAAAAAACCTCCCACACCTCCCCCTGAACCTGAAACATAAAATGAATGCAATTGTTGTTGTTAACTTGTTTATTGCAGCTTATAATGGTTACAAATAAAGCAATAGCATCACAAATTTCACAAATAAAGCATTTTTTTCACTGCATTCTAGTTGTGGTTTGTCCAAACTCATCAATGTATCTTACTGGGTCTCACACCATCCAGACCCTCGCTAAGCTTAGAGGCCGCCATCTACGTGGAATCAGGTTGCTGTCAGGGTGTGTGGGAGGCTCTGGTTTCCTGAGAAACCATCTGATCCTGCATTCAAAGTCATTCCTCTCTGGCTACTTCACTCTGCACATGAGAGATGCTCAGACTGTATCAAGTACTCAGAAAGAAGAGACTACCGGACACTCCTGAATCCAGCTCATGTACAGAACCATCTGAAACACCCAGTGGACCACAATGCTCTGTGGTATGTGAATTTCGATCATCATAGAAGGTGACCCCTCTCTATGTAGAATTTTTATTTTTCAAGCAAATACTTATGACCTCATCAAAGAAAATAATGTCACTTGTTAAATTCACTGTAGTGATACATAAAGTAAATGCTACCTCTGACCTCTGACCCAGTCACCTTCTGTAGAGAGTTCCAGTCCTTTTGTGATGGAATACATCATTTCCAACTTAAAACTTTCACCTTGAAGTTATGGTCTAGTTAAGACATCAGGGGCACCTCCGTTTCTTGGTTTTGTATTGTTTGAAAGAAGACGACATCTTCCTCCTTAGCTGTGTGTTG-AAATGAA-GG

Animal ID 121DF

GCTACATCAACAGGCGGATCTAAATGCATCAGCCATACCACATTTGTAGAGGTTTTACTTGCTTTAAAAAACCTCCCACACCTCCCCCTGAACCTGAAACATAAAATGAATGCAATTGTTGTTGTTAACTTGTTTATTGCAGCTTATAATGGTTACAAATAAAGCAATAGCATCACAAATTTCACAAATAAAGCATTTTTTTCACTGCATTCTAGTTGTGGTTTGTCCAAACTCATCAATGTATCTTACTGGGTCTCACACCATCCAGACCCTCGCTAAGCTTAGAGGCCGCCATCTACGTGGAATCAGGTTGCTGTCAGGGTGTGTGGGAGGCTCTGGTTTCCTGAGAAACCATCTGATCCTGCATTCAAAGTCATTCCTCTCTGGCTACTTCACTCTGCACATGAGAGATGCTCAGACTGTATCAAGTACTCAGAAAGAAGAGACTACCGGACACTCCTGAATCCAGCTCATGTACAGAACCATCTGAAACACCCAGTGGACCACAATGCTCTGTGGTATGTGAATTTCGATCATCATAGAAGGTGACCCCTCTCTATGTAGAATTTTTATTTTTCAAGCAAATACTTATGACCTCATCAAAGAAAATAATGTCACTTGTTAAATTCACTGTAGTGATACATAAAGTAAATGCTACCTCTGACCTCTGACCCAGTCACCTTCTGTAGAGAGTTCCAGTCCTTTTGTGATGGAATACATCATTTCCAACTTAAAACTTTCACCTTGAAGTTATGGTCTAGTTAAGACATCAGGGGCACCTCCGTTTCTTGGTTTTGTATTGTTTGAAAGAAGACGACATCT-CCTCCTTAGCTGTGTGTTGAAAATGAAA-G

***Drd2*-Cre raw sequence reads**

Across 5’ homology arm into Cre:

Animal ID 245DF

GGAGCAAACTGGAGTTATAGATATGGATGCTGAGCACAGTATCTATTATTTCTTTTAGAACGTAAGATACAAAACCATGTGTATTTTGTTTGCCTGTGTTTTTCATGTAAAATGATGATTTAATAAATGTTAAAAAAAATCAAGAATTTGTGGATGCCTATTTCTCTTCTACCACAAGACTTTGGAACTCCTAACTAACTGTGCACATGAGTGGGTTGGACAAGTGAAAGAATGGATGAATAGCTCTTTTGAAGCTTATTTATGTTATTAGGCTTGAAAATGTATAGTTTTCACTTATTGAACCATCTGCTAGAAGAACAGAAGCTTGCCCCAGCATTATAAGATCATTGGCTATGGGGAAAAAAAACTATAGTTTGGAGAATTCTCTACTCTGCTAGCTAGCTCTTGGGTTTTCTTCAGGGAACCCTCTTTGAGAGGAAGCATGCCTTGAAAGTGCTCCTACTCACTCCATGTGTTCATTTCTCCTGGCCAGAGCCGTGCCACCCAGTGGCCCCACTGCCCCAAATGTCCAATTTACTGACCGTACACCAAAATTTGCCTGCATTACCGGTCGATGCAACGA-TG

Animal ID 246DF

GAGCAACTGGAGTTATAGATATGGATGCTGAGCACAGTATCTATTATTTCTTTTAGAACGTAAGATACAAAACCATGTGTATTTTGTTTGCCTGTGTTTTTCATGTAAAATGATGATTTAATAAATGTTAAAAAAAATCAAGAATTTGTGGATGCCTATTTCTCTTCTACCACAAGACTTTGGAACTCCTAACTAACTGTGCACATGAGTGGGTTGGACAAGTGAAAGAATGGATGAATAGCTCTTTTGAAGCTTATTTATGTTATTAGGCTTGAAAATGTATAGTTTTCACTTATTGAACCATCTGCTAGAAGAACAGAAGCTTGCCCCAGCATTATAAGATCATTGGCTATGGGGAAAAAAAACTATAGTTTGGAGAATTCTCTACTCTGCTAGCTAGCTCTTGGGTTTTCTTCAGGGAACCCTCTTTGAGAGGAAGCATGCCTTGAAAGTGCTCCTACTCACTCCATGTGTTCATTTCTCCTGGCCAGAGCCGTGCCACCCAGTGGCCCCACTGCCCCAAATGTCCAATTTACTGACCGTACACCAAAATT-GCCTGCATTACCGGTCGATGCAACGAGT

Animal ID 247DF

AACTGGAGTTATAGATATGGATGCTGAGCACAGTATCTATTATTTCTTTTAGAACGTAAGATACAAAACCATGTGTATTTTGTTTGCCTGTGTTTTTCATGTAAAATGATGATTTAATAAATGTTAAAAAAAATCAAGAATTTGTGGATGCCTATTTCTCTTCTACCACAAGACTTTGGAACTCCTAACTAACTGTGCACATGAGTGGGTTGGACAAGTGAAAGAATGGATGAATAGCTCTTTTGAAGCTTATTTATGTTATTAGGCTTGAAAATGTATAGTTTTCACTTATTGAACCATCTGCTAGAAGAACAGAAGCTTGCCCCAGCATTATAAGATCATTGGCTATGGGGAAAAAAAACTATAGTTTGGAGAATTCTCTACTCTGCTAGCTAGCTCTTGGGTTTTCTTCAGGGAACCCTCTTTGAGAGGAAGCATGCCTTGAAAGTGCTCCTACTCACTCCATGTGTTCATTTCTCCTGGCCAGAGCCGTGCCACCCAGTGGCCCCACTGCCCCAAATGTCCAATTTACTGACCGTACACCAAAATTTGCCTGCATTAC-GGTCGATGCAAAC

Out of bGH poly(A) across 3’ homology arm:

Animal ID 245DF

GTAGGTGTCATTCTATTCTGGGGGTGGGGTGGGGCAGGACAGCAAGGGGGAGGATTGGGAAGACAATAGCAGGCATGCTGGGGATGCGGTGGGCTCTATGGTGGATCCACTGAACCTGTCCTGGTACGATGACGATCTGGAGAGGCAGAACTGGAGCCGGCCCTTCAATGGGTCAGAAGGGAAGGCAGACAGGCCCCACTACAACTACTATGCCATGCTGCTCACCCTCCTCATCTTTATCATCGTCTTTGGCAATGTGCTGGTGTGCATGGCTGTATCCCGAGAGAAGGCTTTGCAGACCACCACCAACTACTTGATAGTCAGCCTTGCTGTGGCTGATCTTCTGGTGGCCACACTGGTAATGCCGTGGGTTGTCTACCTGGAGGTAGGTCTGGGGCCCTTACTTCAAGAACCTTAGACTGGAGTCTGTATTCTTAAGCCCCCAGCAGTTTCTGCCTGTTCCCTTGAGTAGCCTTTCCCTCTCTGCTGGACTCAGTTTCTCTCTTTATGGAGCAGACAGTGATCATAATCATGACTGTGTGCTTTACAGTGTGCTTCCTAAGTACAGGGTCCACGGTAAGTATTCTATACACAGAATCTTCCTATATGATCCTCATAGCAGCTCTGGGGACAGAACT-GCA

Animal ID 246DF

TGAGTAGGTGTCATTCTATTCTGGGGGGTGGGGTGGGGCAGGACAGCAAGGGGGAGGATTGGGAAGACAATAGCAGGCATGCTGGGGATGCGGTGGGCTCTATGGTGGATCCACTGAACCTGTCCTGGTACGATGACGATCTGGAGAGGCAGAACTGGAGCCGGCCCTTCAATGGGTCAGAAGGGAAGGCAGACAGGCCCCACTACAACTACTATGCCATGCTGCTCACCCTCCTCATCTTTATCATCGTCTTTGGCAATGTGCTGGTGTGCATGGCTGTATCCCGAGAGAAGGCTTTGCAGACCACCACCAACTACTTGATAGTCAGCCTTGCTGTGGCTGATCTTCTGGTGGCCACACTGGTAATGCCGTGGGTTGTCTACCTGGAGGTAGGTCTGGGGCCCTTACTTCAAGAACCTTAGACTGGAGTCTGTATTCTTAAGCCCCCAGCAGTTTCTGCCTGTTCCCTTGAGTAGCCTTTCCCTCTCTGCTGGACTCAGTTTCTCTCTTTATGGAGCAGACAGTGATCATAATCATGACTGTGTGCTTTACAGTGTGCTTCCTAAGTACAGGGTCCACGGTAAGTATTCTATACACAGAATCTTCCTATATGATCCTCATAGCAGCTCTGGG-ACAGAACTGGCACTGTCA

Animal ID 247DF

ATTTGTCTGAGTAGGTGTCATTCTATTCTGGGGGGTGGGGTGGGGCAGGACAGCAAGGGGGAGGATTGGGAAGACAATAGCAGGCATGCTGGGGATGCGGTGGGCTCTATGGTGGATCCACTGAACCTGTCCTGGTACGATGACGATCTGGAGAGGCAGAACTGGAGCCGGCCCTTCAATGGGTCAGAAGGGAAGGCAGACAGGCCCCACTACAACTACTATGCCATGCTGCTCACCCTCCTCATCTTTATCATCGTCTTTGGCAATGTGCTGGTGTGCATGGCTGTATCCCGAGAGAAGGCTTTGCAGACCACCACCAACTACTTGATAGTCAGCCTTGCTGTGGCTGATCTTCTGGTGGCCACACTGGTAATGCCGTGGGTTGTCTACCTGGAGGTAGGTCTGGGGCCCTTACTTCAAGAACCTTAGACTGGAGTCTGTATTCTTAAGCCCCCAGCAGTTTCTGCCTGTTCCCTTGAGTAGCCTTTCCCTCTCTGCTGGACTCAGTTTCTCTCTTTATGGAGCAGACAGTGATCATAATCATGACTGTGTGCTTTACAGTGTGCTTCCTAAGTACAGGGTCCACGGTAAGTATTCTATACACAGAATCTTCCTATATGATCCTCATAGCAGCTCTGGGGACAGAACTG-CACTG

***Triml2*-P2A-Cre raw sequence reads**

Across 5’ homology arm and P2A into Cre:

Animal ID 752DA

TCCCGAAGACCTGAGAAGTGTGGGATTCCGAGAGACACCAAAGACTGGGCCTGGCACTACAAGGAGATTGGATTTCAGCGCTTCTGTGTTGGGTGCAGAGAGCTTCACCACGGGGAGGCATTATTGGGAGGTGGCTGTGGGACAGGCAACCCAATGGCAGGTGGGCATATGTGACTGTACAGAGAGAAAGGACAACATTCCCGGGGCTTCTGGAGATAAAGTCTTGCTCATGGGGTCCATGATGGGGACCGATTGTACCCTCTGGGTCTTTCCCCCTTTAAGAAAGGTCTGTCTGAGAAACCAAATGTACAAAGTTGGAGTCTTCCTAGACTGTGAATGTGGGCAAGTATCCTTCTACAATGTGACAGAGCAGTCCCTCATTTACAGTTTCTCTGACCTTACCTTCCGAGGAGCGATTAAACCAATATTTTCTCTTTGTATTCCAAATGGAGACATGAGTTCAGACTCTCTCACTGTCTGTCTTCCTCAGACTCATCCCGGAAGCGGAGCTACTAACTTCAGCCTGCTGAAGCAGGCTGGAGACGTGGAGGAGAACCCTGGACCTGCTAGCATGGCCAATTTACTGACCGTACACCAAAATTTTGCCTGCATTACCGGTCGATGCA-CGAGTGAT

Animal ID 756DA

AGACCTGAGAAGTGTGGGATTCCGAGAGACACCAAAGACTGGGCCTGGCACTACAAGGAGATTGGATTTCAGCGCTTCTGTGTTGGGTGCAGAGAGCTTCACCACGGGGAGGCATTATTGGGAGGTGGCTGTGGGACAGGCAACCCAATGGCAGGTGGGCATATGTGACTGTACAGAGAGAAAGGACAACATTCCCGGGGCTTCTGGAGATAAAGTCTTGCTCATGGGGTCCATGATGGGGACCGATTGTACCCTCTGGGTCTTTCCCCCTTTAAGAAAGGTCTGTCTGAGAAACCAAATGTACAAAGTTGGAGTCTTCCTAGACTGTGAATGTGGGCAAGTATCCTTCTACAATGTGACAGAGCAGTCCCTCATTTACAGTTTCTCTGACCTTACCTTCCGAGGAGCGATTAAACCAATATTTTCTCTTTGTATTCCAAATGGAGACATGAGTTCAGACTCTCTCACTGTCTGTCTTCCTCAGACTCATCCCGGAAGCGGAGCTACTAACTTCAGCCTGCTGAAGCAGGCTGGAGACGTGGAGGAGAACCCTGGACCTGCTAGCATGGCCAATTTACTGACCGTACACCAAATTTGCCTGCATTACCGGTCGATGCAAC

Animal ID 758DA

TAGTCTTATCCGAAGACCTGAGAAGTGTGGGATTCCGAGAGACACCAAAGACTGGGCCTGGCACTACAAGGAGATTGGATTTCAGCGCTTCTGTGTTGGGTGCAGAGAGCTTCACCACGGGGAGGCATTATTGGGAGGTGGCTGTGGGACAGGCAACCCAATGGCAGGTGGGCATATGTGACTGTACAGAGAGAAAGGACAACATTCCCGGGGCTTCTGGAGATAAAGTCTTGCTCATGGGGTCCATGATGGGGACCGATTGTACCCTCTGGGTCTTTCCCCCTTTAAGAAAGGTCTGTCTGAGAAACCAAATGTACAAAGTTGGAGTCTTCCTAGACTGTGAATGTGGGCAAGTATCCTTCTACAATGTGACAGAGCAGTCCCTCATTTACAGTTTCTCTGACCTTACCTTCCGAGGAGCGATTAAACCAATATTTTCTCTTTGTATTCCAAATGGAGACATGAGTTCAGACTCTCTCACTGTCTGTCTTCCTCAGACTCATCCCGGAAGCGGAGCTACTAACTTCAGCCTGCTGAAGCAGGCTGGAGACGTGGAGGAGAACCCTGGACCTGCTAGCATGGCCAATTTACTGACCGTACACCAAAATTTGCCTGCATTACCG-TCGATGCAACGAGTGATGA

Out of Cre across SV40 poly(A) and 3’ homology arm:

Animal ID 752DA

TTGTCCAAACTCATCAATGTATCTTAAATGCTGCTGATAGCTCTTCATCTTCCTGTGTGTGGAACCCAAGATCAAAAGAGACACAAAAATAAGATCCAGACTTTGTGAACACCACTTATGTAATTAATCTAATATAATCCAATAAAGCCATTAGACTTAATGTGGCTCTCTGCATGTGTTCAGTAGAAGGAGCTCTGTTTAAGTTCAGTGATGATGGGACTCAGGGGATCAAGGCACTTACAGACAATGCAGATAGCCTGAGTTCAATTCCTGGACCTCACAGGAGAGAGAAAGAACAGACTTCTGAAAGTTTTCCTCTGACATTCACATGCATAATGTGGCAAGTGGCTATTGTTCCACTAAAACAATAAAGTACAATAGAAAACAAGGCAATAATGAAGGGTTGTAACATTGAATATAATACACACACACATTCACATATATACATGAGATGTATCATATATTTGCTCAATATATATTTGTGTGTGTGTGTGTGTGTGTGTGTGTGT

Animal ID 756DA

GTTTGTCCAAACTCATCAATGTATCTTAAATGCTGCTGATAGCTCTTCATCTTCCTGTGTGTGGAACCCAAGATCAAAAGAGACACAAAAATAAGATCCAGACTTTGTGAACACCACTTATGTAATTAATCTAATATAATCCAATAAAGCCATTAGACTTAATGTGGCTCTCTGCATGTGTTCAGTAGAAGGAGCTCTGTTTAAGTTCAGTGATGATGGGACTCAGGGGATCAAGGCACTTACAGACAATGCAGATAGCCTGAGTTCAATTCCTGGACCTCACAGGAGAGAGAAAGAACAGACTTCTGAAAGTTTTCCTCTGACATTCACATGCATAATGTGGCAAGTGGCTATTGTTCCACTAAAACAATAAAGTACAATAGAAAACAAGGCAATAATGAAGGGTTGTAACATTGAATATAATACACACACACATTCACATATATACATGAGATGTATCATATATTTGCTCAATATATATTTGTGTGTGTGTGTGTGTGTGTGTGTGTGTGTGTCATACCTATACATCACATACATGGTCAGTATACATAATACGTGTGTGTATATATATGTGATGTATATGATCGGAAAGTTAGAATATATGAATACATTCTTGTT-CGAAAGACCTAGAA

Animal ID 758DA

TCATCAATGTATCTTAAATGCTGCTGATAGCTCTTCATCTTCCTGTGTGTGGAACCCAAGATCAAAAGAGACACAAAAATAAGATCCAGACTTTGTGAACACCACTTATGTAATTAATCTAATATAATCCAATAAAGCCATTAGACTTAATGTGGCTCTCTGCATGTGTTCAGTAGAAGGAGCTCTGTTTAAGTTCAGTGATGATGGGACTCAGGGGATCAAGGCACTTACAGACAATGCAGATAGCCTGAGTTCAATTCCTGGACCTCACAGGAGAGAGAAAGAACAGACTTCTGAAAGTTTTCCTCTGACATTCACATGCATAATGTGGCAAGTGGCTATTGTTCCACTAAAACAATAAAGTACAATAGAAAACAAGGCAATAATGAAGGGTTGTAACATTGAATATAATACACACACACATTCACATATATACATGAGATGTATCATATATTTGCTCAATATATATTTGTGTGTGTGTGTGTGTGTGTGTGTGTGTGTG-CA
